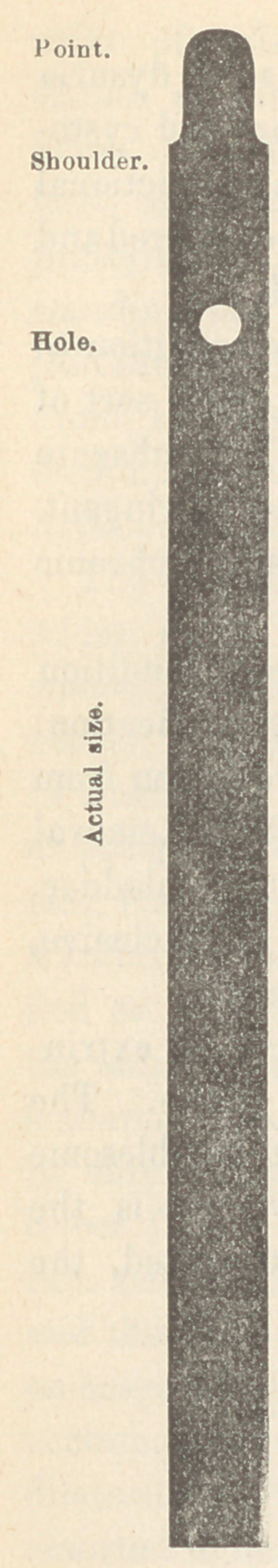# New Umbilical Cord Repositor

**Published:** 1881-11

**Authors:** P. O’Connell

**Affiliations:** Sioux City, Iowa


### ARTICLE III.

NEW UMBILICAL CORD REPOSITOR. By P. O'CONNELL, M.D., Sioux City, Iowa.

The accompanying drawing represents the tip, actual size, of an instrument intended as a repositor of prolapsed funis. This simple device is made of a smooth piece of flat whalebone, sixteen or eighteen inches long, one-fourth of an inch wide, and about half a line thick, with the sharp edges rounded off.

Actual size.

One inch from the top, a hole one-eighth of an inch diameter, is drilled through the whalebone. Half-way between the hole and the point, the edges are cut away a little, making a slight shoulder; the point is nicely rounded, yet not at all sharp.

These proportions make an instrument sufficiently strong to carry the cord up beyond the presenting part, yet flexible as well as thin enough to be left *in utero*, without doing harm, until the presentation has advanced far enough to prevent any subsequent descent of the funis ; or even until the completion of the second stage of labor when it will be expelled with the child.

The instrument is quickly and easily applied thus: Take a piece of narrow tape or soft twine, at least twice the length of the whalebone ; double it, and pass the double through the hole. Pass the point of the instrument *under* the funis; make a snare-loop of the doubled string passed through the hole, and slip it on the point down to the shoulder, *over* the funis, drawing the snare-loop pretty tightly on the point of the whalebone. The free ends of the tape are drawn just tight enough to hold the funis snugly without diminishing the circulation in it, and loosely wound round the instrument a few times and held from slipping through the hole while the cord is being replaced. The funis, lying on the flat surface of the instrument between the hole and shoulder, is kept there in place by the string over it, and is then pushed up into the uterus beyond the presenting part, the whole process being completed in one or two minutes.

The slight shoulder near the top of the whalebone prevents the snare-loop on the tape running down too far and compressing the cord too much. By loosening the free ends of the tape a little, and rotating the instrument at the same time that slight traction is made on it alone, the snare-loop will slip off the point, freeing the funis, when the whalebone and tape are withdrawn from the uterus. The advantages of this little device are, that the funis can be replaced quickly, and with certainty, and without placing the woman in the awkward genu-pectoral posture as usually advised.

Any one can make the instrument for himself, as I have done, in an hour. Whalebone of the proper width and thickness can be purchased at any dry-goods establishment, and only needs finishing.

## Figures and Tables

**Figure f1:**